# Stochastically modeling multiscale stationary biological processes

**DOI:** 10.1371/journal.pone.0226687

**Published:** 2019-12-26

**Authors:** Michael A. Rowland, Michael L. Mayo, Edward J. Perkins, Natàlia Garcia-Reyero

**Affiliations:** Environmental Laboratory, U.S. Army Corps of Engineers, Vicksburg, MS, United States of America; King’s College London, UNITED KINGDOM

## Abstract

Large scale biological responses are inherently uncertain, in part as a consequence of noisy systems that do not respond deterministically to perturbations and measurement errors inherent to technological limitations. As a result, they are computationally difficult to model and current approaches are notoriously slow and computationally intensive (multiscale stochastic models), fail to capture the effects of noise across a system (chemical kinetic models), or fail to provide sufficient biological fidelity because of broad simplifying assumptions (stochastic differential equations). We use a new approach to modeling multiscale stationary biological processes that embraces the noise found in experimental data to provide estimates of the parameter uncertainties and the potential mis-specification of models. Our approach models the mean stationary response at each biological level given a particular expected response relationship, capturing variation around this mean using conditional Monte Carlo sampling that is statistically consistent with training data. A conditional probability distribution associated with a biological response can be reconstructed using this method for a subset of input values, which overcomes the parameter identification problem. Our approach could be applied in addition to dynamical modeling methods (see above) to predict uncertain biological responses over experimental time scales. To illustrate this point, we apply the approach to a test case in which we model the variation associated with measurements at multiple scales of organization across a reproduction-related Adverse Outcome Pathway described for teleosts.

## Introduction

Biological processes at all scales and the measurements of their outputs, from the molecular to the population level, are noisy. In order to understand and predict behaviors of complex biological systems it is essential to include stochasticity into models in order to capture empirically-observed variability. For example, a population of isogenic cells can exhibit a wide array of responses to the same environment as a result of the molecular noise caused by proteins diffusing to reach target regulated gene or in the variability observed in intercellular biochemistry [1–7]. The technology used to measure the biological responses can introduce measurement error, including laboratory and batch effects [8–10]. On larger scales, researchers have come to appreciate the necessity of stochastic modeling in predicting the movement of animal populations traditionally assumed as deterministic or mechanistic [11–14]. A greater appreciation for the variability inherent to biological phenomena has led to an increasing use of stochastic models, which are typically harder to fit with sparse datasets and are more computationally expensive to execute.

A recurring issue in the mathematical modeling of noisy biological phenomena is what is known colloquially as the “parameter identification problem.” Biological models often admit many sets of parameter values consistent with the training data, inviting questions as to why certain values were chosen over others, especially when parameter choice affects model performance such as sensitivity [15]. These considerations have spurred further research into new methods of parameter estimation and model development [16–18].

In this work, we seek to avoid the parameter identification problem by embracing the uncertainty associated with fitted parameter values, rather than justifying the choice of a singular set of values post hoc. In what follows we describe a data-driven approach to modeling developed from the premise that parameter values estimated from curve-fitted models are but one parameter set sampled from a distribution of all sets that are consistent with data. The output permits reconstruction of the conditional probability distribution between an input value (e.g., exposure concentration in a toxicity experiment) and a biological endpoint (e.g., animal mortality). Briefly, the method works by associating experimental data for endpoints represented by adjacent nodes of a hierarchical network with one root and one leaf, fitting the data to a set of sigmoidal curves, and bootstrapping the regression to obtain confidence intervals that can then be used to estimate the distribution of sigmoid parameter values in each level of the hierarchy. The conditional endpoint distribution may be found through multiple instances of propagating an input value through the network based on the now probabilistic relationships between adjacent nodes. The comparison of the probability density clouds produced by the model with the experimental data highlight either the modeling misspecification (i.e. a modeled linear relationship in place of a sigmoidal) or potential errors in the data. The method is illustrated with a biological pathway that results in a decrease in reproductive output, or an Adverse Outcome Pathway (AOP), with experimental data obtained from fathead minnow (*Pimephales promelas*). The AOP framework is an increasingly accepted approach to link biological pathways and other non-traditional toxicological data to adverse outcomes [19]. This framework also provides a useful construct in which to develop predictive models for chemical hazard [20]. The AOP covers multiple scales of events across multiple timescales, from the (ant)agonism of receptors (molecular), to the blood plasma concentrations of small molecules (physiological), to the average fertility of a population of organisms; simulating such a pathway demonstrates the utility of this approach as a multiscale platform. Here, we demonstrate our approach using a network of AOPs related to fathead minnow reproduction.

## Methods

### Correlated pathway modeling

We are interested in modeling the correlated relationship between the continuous quantities of an experimental input, *X*, and an associated measureable output, *Y*. For simplicity, we will assume that *X* is correlated to *Y* through a linear chain of *n* intermediate variables: {*Y*_*i*_:*i* = 1,2,…,*n*}. We model the input, output, and intermediate quantities as random variables, and use the standard notation which distinguishes between a random variable *X* and one of its possible values, *X* = *x*. Our goal, then, is to obtain the joint probability, *p*(*x*,*y*)*dxdy*, between the input and output, which encodes the variance associated with noise of the underlying stochastic processes in addition to experimental and measurement uncertainty. Our primary modeling proposition is that this joint distribution can be decomposed by means of a Markovian assumption associated with the intermediate stochastic processes:

$$p(y,x)=\int_{{y_{i}\in Y}_{i}}\cdots \int_{y_{1}\in Y_{1}}dy_{i}\cdots dy_{1}\;p(y|y_{i})p(y_{i}|y_{i-1})\cdots p(y_{1}|x).$$

Although this description is applicable to linear chains, it can be extended to more complex networks of correlated associations through calculation of the appropriate conditional probability distributions.

### Bootstrapping the interaction regressions

**Fig 1:**
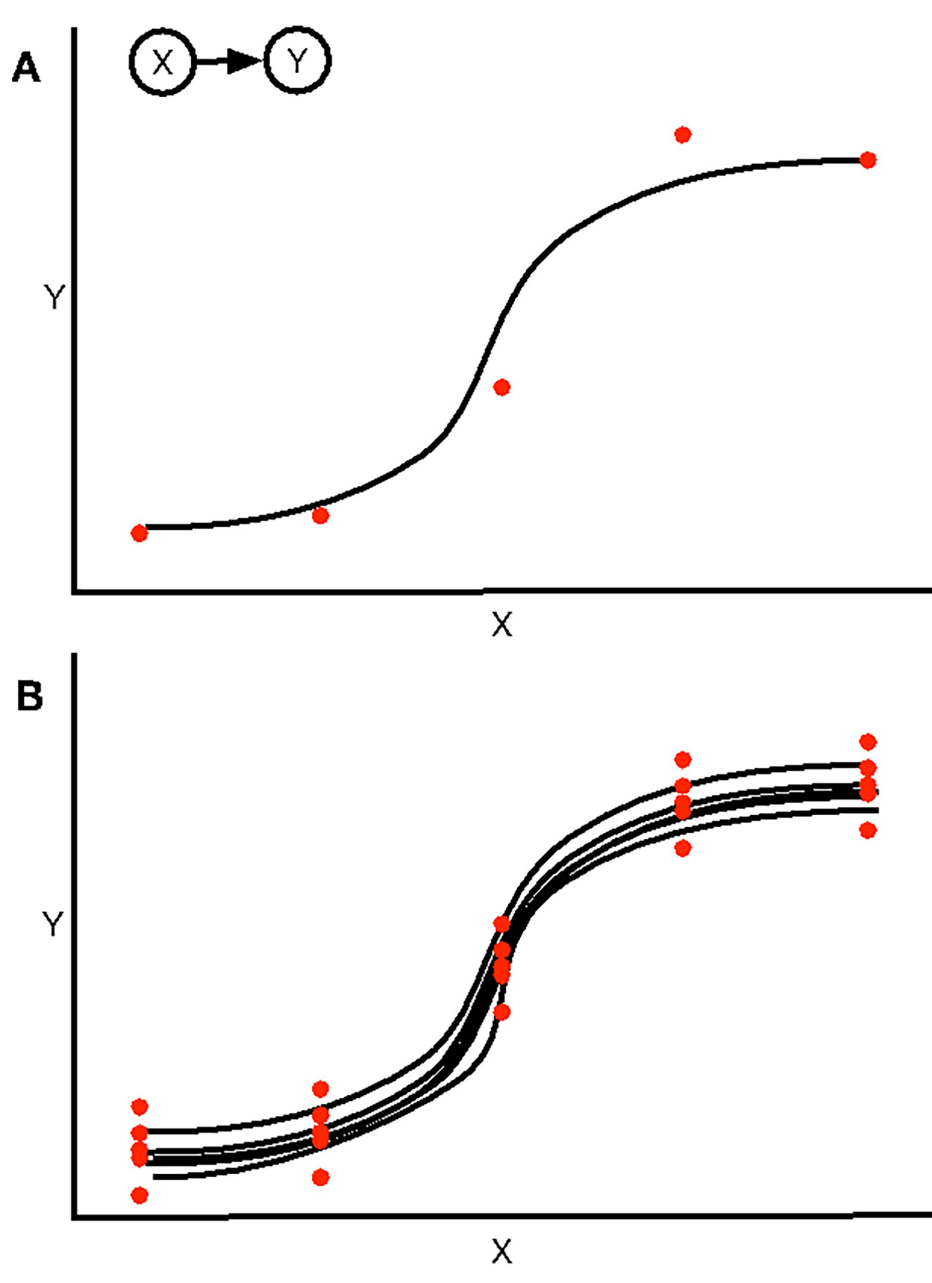
Example of the algorithm. (A) Consider an interaction between two nodes, X and Y, with measured values of Y in response to various activities of X presented by the red dots. The sigmoidal curve in black then represents the average response of Y to increasing X. (B) The algorithm assumes that the shape of the curve is correct, but that the assignment of the residuals is not. The red dots represent the potential values of responses of Y to X, determined by adding each of the residuals to the average response obtained in panel A. For each bootstrap, the algorithm selects one of these values for each value of X and fits another sigmoid. Analyzing the values of the coefficients for the sigmoids across n = 1000 bootstraps provides the 95% confidence intervals for each coefficient. These confidence intervals are then used to stochastically simulate the interaction between X and Y.

Our goal is to reconstruct the relevant conditional distributions, *p*(*y*_*i*_|*y*_*i*−1_), from partial sets of experimental data. To begin, we conceptualize the aforementioned random variables as nodes of a network determined through examination of bulk molecular data, such as protein phosphorylation within a cell culture and hormone concentrations in blood plasma, or aggregate data from individual organisms, such as the number of eggs spawned from individual fish. It is often the case, though, that few paired measurements may be available for the nodes and, even if many measurements were available, it would be noisy, due to biological noise or measurement errors occurring during experiments [3, 8–10]. Assuming that there is an obvious average mathematical relationship between the nodes, a curve fit to the data would still provide only an approximation of the data (**Fig 1A**).

We account for the noise in the data by bootstrapping the regressions. If we had a dataset of paired experimental values {(*x*_*i*_,*y*_*i*_),*i* = 1,…,*n*}, for nodes *X* and *Y*, we can then fit the function *f*(*x*,{*c*}) to this data, in which {*c*} is the set of coefficients necessary for *f*. This function would be akin to the black sigmoid presented in **Fig 1A**. The algorithm determines the 95% confidence intervals of the coefficients {*c*} by resampling the residuals with replacement [21]. The set of residuals of this regression can then be calculated: {*r*_*i*_ = *y*_*i*_−*f*(*x*_*i*_,{*c*})}. For each bootstrap, we determine a new set of values $\left\{{\hat{y}}_{i}=f(x_{i},\{c\})+r_{j}\right\}$ in which *r*_*j*_∈{*r*_*i*_} is a randomly selected value from the set of residuals. Essentially the bootstrapping assumes that the error presented by the residuals is not specific to that particular point; there are *n* values of ${\hat{y}}_{i}$ from which the bootstrapping procedure can choose (**Fig 1B**, red dots). We can then fit the function $\hat{f}(x,\{\hat{c}\})$ to the set of paired values ${\{(x}_{i},{\hat{y}}_{i}),i=1,\ldots ,n\}$ (**Fig 1B**, black curves). This is repeated *m* times; in this work *m* = 999. For every coefficient *c*, the original and bootstrapped values are sorted into ascending order, and the *c*_(*m*+1)*0.025_ and *c*_(*m*+1)*0.975_ values are taken as the estimates of the lower and upper bounds of the 95% confidence interval for the coefficient.

### Stochastic simulations using bootstrapped interactions

For each simulation of the model, our approach chooses values for the coefficients for each interaction in the network by selecting it at random from the Gaussian distribution underlying the 95% confidence intervals determined by the bootstrapping. Given an input activity, the algorithm propagates the activity through the network based on the randomly parameterized interaction functions. This is then repeated any number of times to obtain a sample distribution of activity levels for every node for the given input. As such, the activity of a node from a single simulation would be analogous to the fold increase in phosphorylation of a protein in response to an input within a single cell while the distribution of activity levels of that node across many simulations would be the fold increase in phosphorylation of the population of cells as measured by Western blotting.

### Thresholds for the impact of an input on the system

A central question to analyzing the results of our simulations is at what activity levels does the input have a significant impact on the output of the system? To address this point, we ran the models for 1000 replicates per activity level across a wide range of input activities. We then sequentially compared each sample population of responses to each activity level to the responses at the lowest and highest levels. The comparisons were executed using the Kolmogorov-Smirnov test by obtaining the cumulative distribution functions (CDFs) of the responses and finding the maximum difference (*D*_*n*,*m*_) between the CDFs. The null hypothesis that the response distributions are drawn from the same distribution is rejected at level *α* = 0.05 if $D_{n,m}>c(\alpha )\sqrt{\frac{n+m}{nm}},c(\alpha )=\sqrt{-\frac{1}{2}ln(\frac{\alpha }{2})}$, where *n* and *m* are the sizes of the two distributions. The first response distributions to reject the null hypothesis as the system moved away from the lowest and highest input activities were taken to be the bounds of the threshold for input activities that significantly impact the system’s response. Below the lower bounds the input has little to no impact on activity when compared to basal while above the upper bounds the system exhibits a response to the input that is significantly different than the basal.

### Data collection

**Fig 2:**
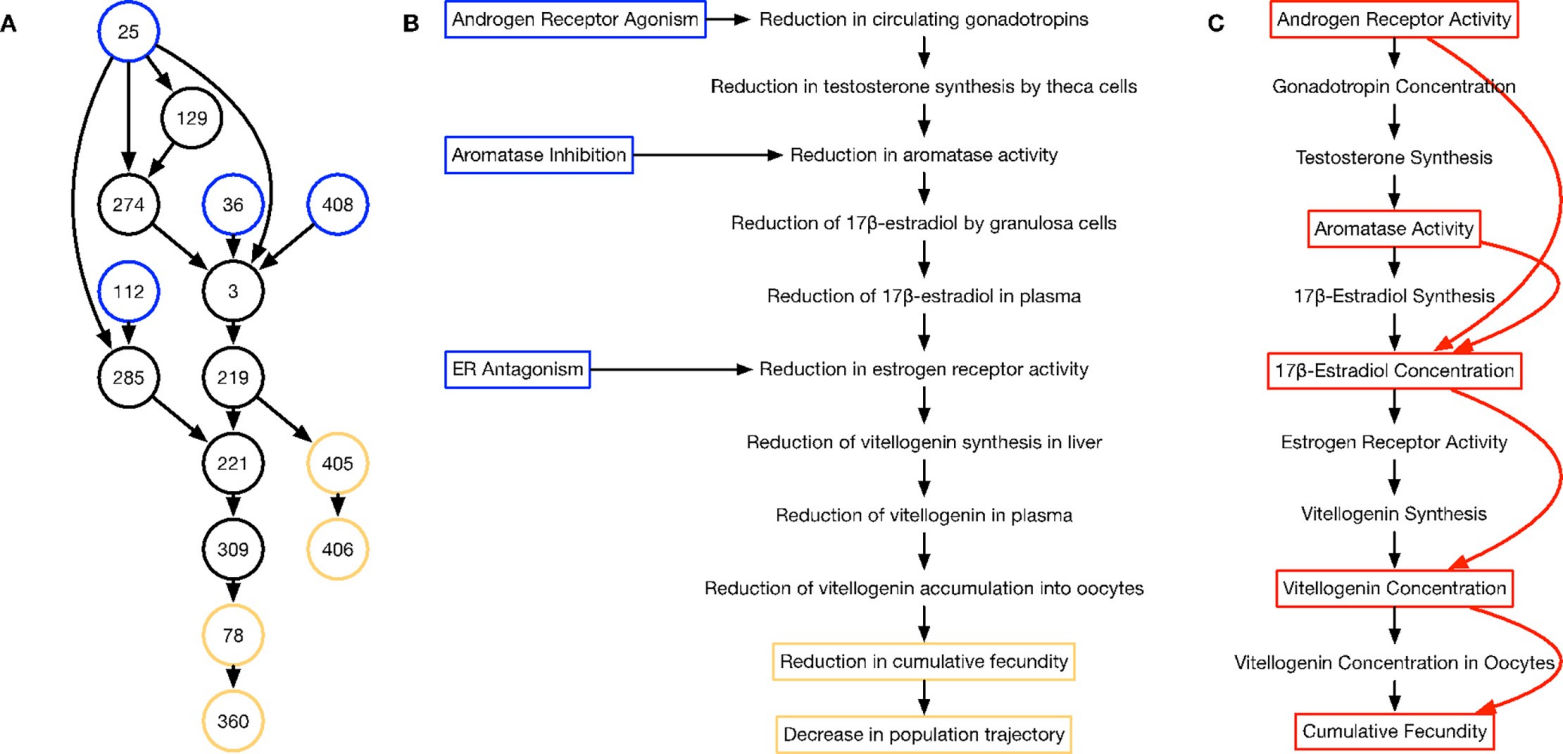
Development of the adverse outcome pathway (AOP) network for female fathead minnow reproductive systems. (A) Diagram of the interactions of various events in the compiled network of 4 AOPs. Blue nodes are molecular initiating events, black nodes are key events, and yellow are adverse outcomes. Numbers represent the indices of the events in the AOPwiki. Information on each node is available at aopwiki.org/events/xxx, where xxx is the index in the node. (B) Diagram of the curated AOP network presented in panel A with indirect interactions removed and events with the same functional consequences collapsed into a single node. (C) Diagram of the quantitative model based on the curated AOP network presented in panel B. Highlighted in red are the nodes for which we have obtained data, with the dose/dose response relationships indicated by red arrows.

In this work we demonstrate the usage and analysis of our approach on a curated network of AOPs related to reproduction in female fathead minnow. The AOP network was compiled from four AOPs available in the AOPwiki (https://aopwiki.org/aops) that highlight the impact of androgen receptor agonism, aromatase inhibition or reduction, or estrogen receptor antagonism with decreasing population trajectories in fathead minnows: “Aromatase (Cyp19a1) reduction leading to impaired fertility in adult female” (AOP:7), “Androgen receptor agonism leading to reproductive dysfunction (in repeat-spawning fish)” (AOP:23), “Aromatase inhibition leading to reproductive dysfunction” (AOP:25), and “Estrogen receptor antagonism leading to reproductive dysfunction (AOP:30)”. The AOPs were combined by overlapping events common to two or more pathways, resulting in a network with several feed-forward structures (**Fig 2A**). This network was manually curated, first by removing indirect edges, such as the relationship between androgen receptor agonism (AOPwiki event ID 25) to reduction in testosterone synthesis by theca cells (AOPwiki event ID 274). Events that have similar biological implications, such as aromatase inhibition (AOPwiki event ID 36) and reduction in aromatase in ovarian granulosa cells (AOPwiki event ID 408), were then merged into a single event. This resulted in the event pathway presented in **Fig 2B**, with a cascade of reductions in activity in response to 3 molecular initiating events, androgen receptor agonism, aromatase inhibition, and estrogen receptor antagonism, leading to a reduction in cumulative fecundity and a decrease in population trajectory.

The events used to define these AOPs, however, are qualitative in nature. In order to simulate this pathway quantitatively, we related the network to a cascade of quantitative activities and concentrations, changes in each of which directly impacts those downstream (**Fig 2C**). We found previously published experimental data relating the impacts of various concentrations of trenbolone α, trenbolone β, prochloraz, fenarimol, and fadrozole on androgen receptor activity, aromatase activity, plasma 17β-estradiol and vitellogenin concentrations, and average fecundity (described in **S1 Table**) [22–27]. We used this dose response data to populate the relationships between several events in the AOP model (**Fig 2C**, highlighted nodes).

## Results

### Predicting reproductive impacts in fathead minnow model of reproduction

AOPs represent linear causal toxicological pathways leading to an adverse outcome of regulatory concern, are useful pathways in which to examine cascading effects across levels of biological complexity and can be combined to create networks and more complex biological models. We compiled a network of AOPs associated with fish reproduction from the AOPwiki, a database of AOPs (https://aopwiki.org/aops) by overlapping events common to each AOP, producing the union of the sets of events (**Fig 2A**). Removing indirect interactions and combining events with similar consequences provided a more transparent, hierarchical network of Key Events (**Fig 2B**) (See Methods for details). The resulting network can be condensed into a “chain” of 11 activities or concentrations, in which parent nodes in the hierarchy affect only the daughter nodes (**Fig 2C**). We found data for androgen receptor (fold change), aromatase activity (fold change), plasma 17β-estradiol concentration (μg/L), plasma vitellogenin concentration (mg/mL), and average fecundity (eggs/female/day) in response to increasing doses of trenbolone α, trenbolone β, prochloraz, fenarimol, and fadrozole. Each of these chemicals are known to directly impact either androgen receptor or aromatase activity (Described in **S1 Table**). We used these data to form an abridged version of the pathway as highlighted in red in **Fig 2C**.

We used the bootstrapping procedure, described in the Methods section, to obtain 95% confidence intervals for the sigmoids:

$$y(x)=y_{max}\frac{{\left(\frac{x}{K}\right)}^{h}}{{1+(\frac{x}{K})}^{h}},\;\mathrm{or}$$ [1]

$$y(x)=\frac{y_{max}}{{1+(\frac{x}{K})}^{h}},$$ [2]

wherein *x* labels the quantifiable activity of the current node (e.g., flux, concentration), *y* labels the predicted activity of an adjacent node, *y*_*max*_ is the maximum observed activity of the next node, *K* is an “equilibrium constant” for the interaction, and *h* is a Hill-type exponent. We determined which function to use with the value of Kendall’s rank correlation coefficient (tau) for the data; a tau > 0 denotes a positive trend in the data, which we modeled using **Eq 1**, while a tau ≤ 0 denotes a negative or unchanging trend in the data, which we modeled using **Eq 2**. The 95% confidence intervals for *K* and *h* for each interaction are presented in **S2 Table**.

**Fig 3:**
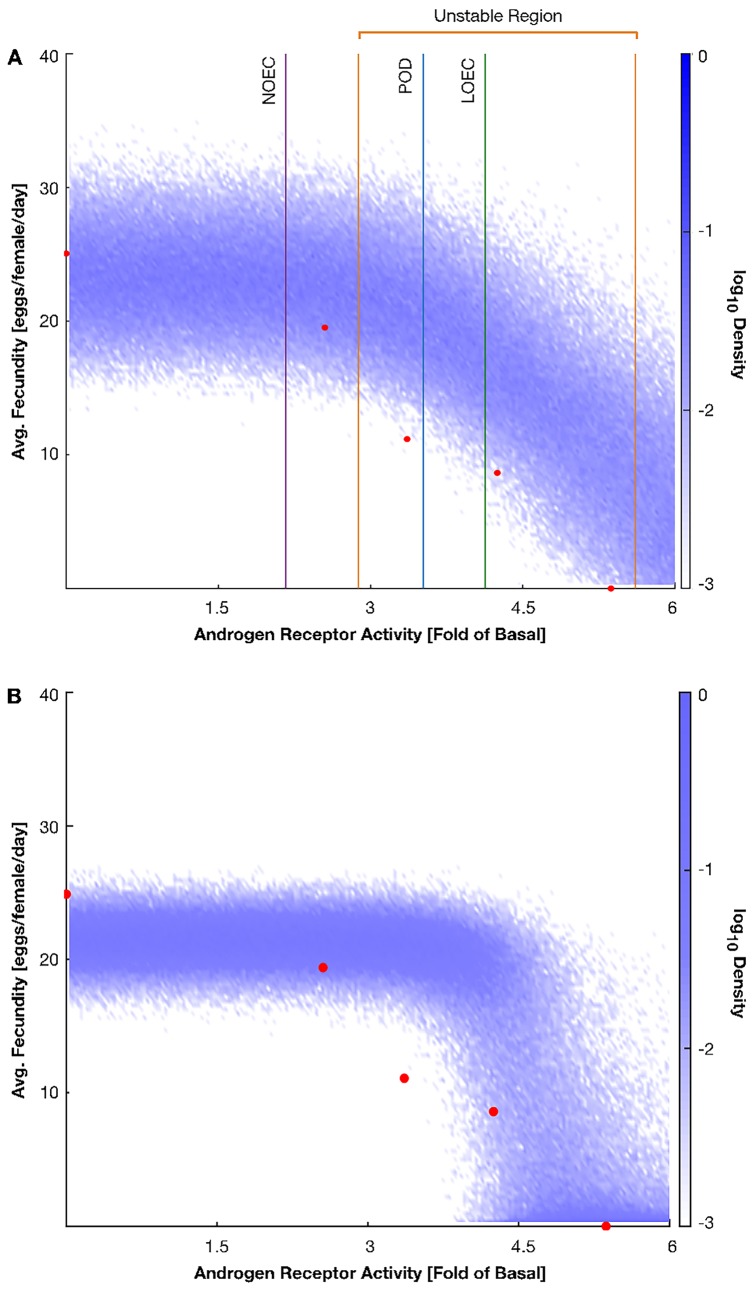
The average fecundity in a group of female fathead minnows in response to increasing levels of androgen receptor activity. The red circles are the average fecundities measured in response to trenbolone α exposures. The shades of blue represent the log_10_ probability of an individual demonstrating a particular average fecundity in response to the indicated androgen receptor activity. The blue probability density clouds were generated using all available experimental data (A) or only data gathered from trenbolone β exposures (B). In (A), the orange vertical lines are the lower and upper bounds of the threshold in which individuals may display a significant decrease in fecundity. The purple, green, and blue vertical lines are the approximate values for the androgen receptor activity in response to the measured No Observable Effect Concentration (NOEC), Lowest Observable Effect Concentration (LOEC), and Point Of Departure (POD) values for trenbolone β [28].

In each model execution, parameter values for each sigmoidal interaction are chosen randomly from a Gaussian distribution defined by the mean and standard deviation calculated from the confidence intervals, with the effects of the input–androgen receptor activity in this example–propagated through the chain. We calculated average fecundity from 1000 simulations per androgen receptor activity level spanning 0- to 6-fold over the basal levels (**Fig 3A**). While the model overpredicts the average fecundity at higher androgen receptor activities, the general behavior of the system remains consistent with an overall decrease in fecundity given an increasing androgen receptor activity.

Thresholds in the androgen receptor activity level mark the first statistically significant difference between fecundity distributions at either end of the data range and androgen receptor activity levels elsewhere as determined via the Kolmorgov-Smirnov test (**Fig 3A,** See Methods for details). At androgen receptor activities below the lower threshold (2.88-fold change) the model predicts no appreciable effect on fecundity, whereas for activities above the higher threshold (5.55-fold change), the model predicts a strong impact on fecundity. The region between these thresholds in androgen receptor activity level reflects a state of weak yet statistically significant impact on fecundity. In principle, the size of the impact (i.e., no, weak, or strong impact) depends on factors that drive fluctuations in the measurements of the Key Events. To determine if either threshold is biologically relevant, we compared them to androgen receptor activities associated with a no observable effect concentration (NOEC), a lowest observable effect concentration (LOEC), and a point of departure (POD) determined for trenbolone β exposed female fathead minnows (**Fig 3A**, purple, green, and blue lines) [28]. The NOEC rests firmly below the lower threshold wherein the model predicts no significant impact on fecundity, whereas the LOEC and POD sit within a region predicting a moderate impact, supporting the biological relevance of the model predictions.

### Impacts of data sparseness, data accuracy, and model specification

One observation to make concerning the fitting of the probability density cloud presented in **Fig 3A** is the over-prediction of the average fecundity by the model for androgen receptor activities above basal (blue vs. red). Given that the model is directly using experimental data to inform the interactions, one might assume that it would accurately predict the experimental data each time. The over-predictions, however, reveal an inherent limitation in this method: the data being inputted into the system must be sufficient to support the type of regression being fit to it. We re-ran the protocol given in the previous section, this time inputting only the response-response data gathered from trenbolone α exposures to demonstrate this limitation in extremis (**Fig 3B**). With a sparser dataset to draw from, the model further over-predicts the average fecundities. This approach, however, is useful in determining interactions for which different regressions or additional data may be necessary to better understand the system being studied.

**Fig 4:**
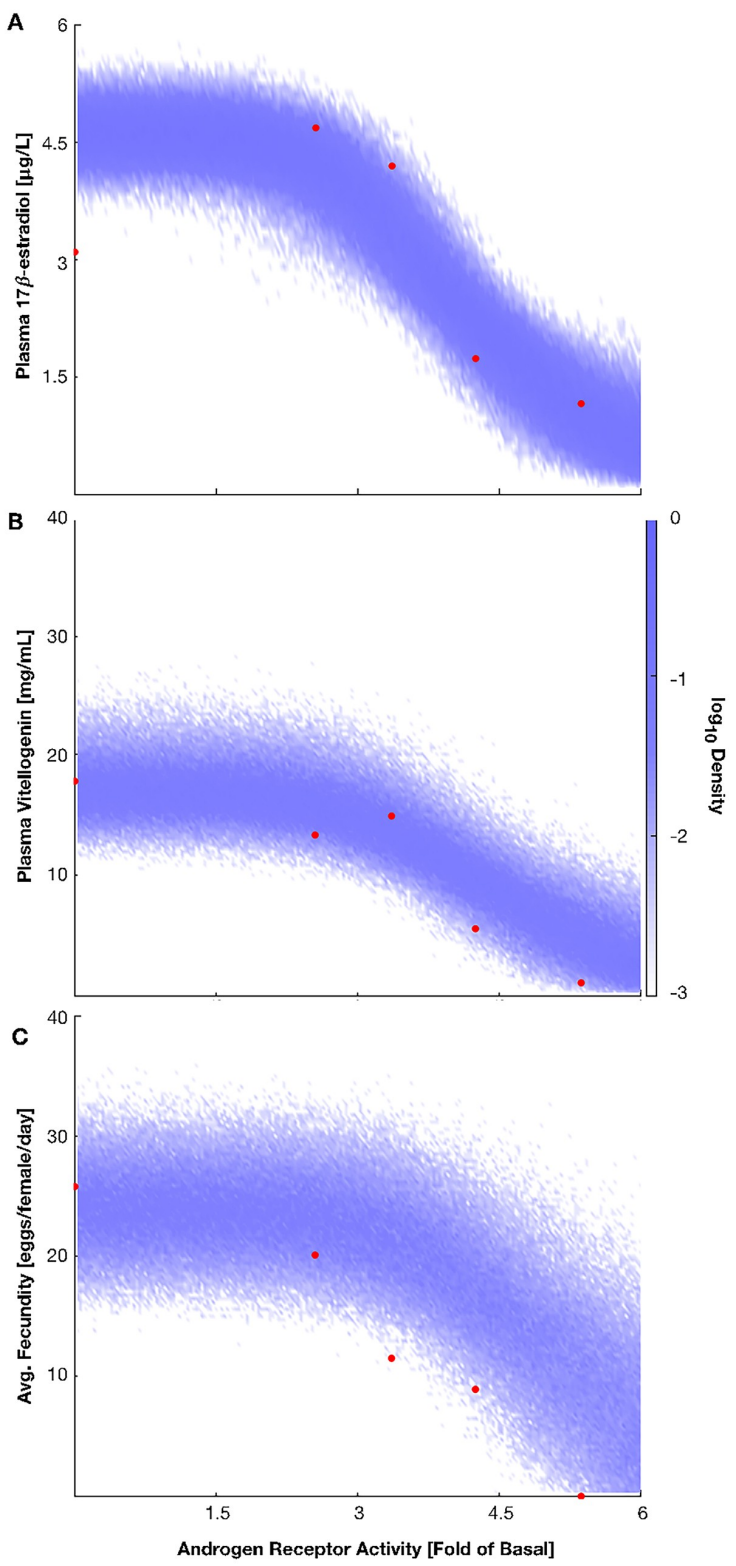
Responses of each step in the reproductive AOP model with respect to increasing levels of androgen receptor activity. Using all available data, probability density clouds for plasma 17β-estradiol concentration (A), plasma vitellogenin concentration (B), and average fecundity (C) were produced for a range of androgen receptor activities. The red dots are the measurements made in response to trenbolone α exposures.

Recall that our approach bootstraps the regressions for multiple interactions, not merely the output vs. input. To illustrate this, we plotted the probability density clouds for plasma 17β-estradiol, plasma vitellogenin, and average fecundity as a functions of the fold change in androgen receptor activity using the full dataset from the previous section (**Fig 4**). The red dots in each panel are the response-response data gathered from the trenbolone-α exposures. Note the initial increase in estradiol concentrations measured in response to smaller increases in androgen receptor activity compared to the reported basal concentration (**Fig 4A**, red). It is likely the disparity between the basal estradiol concentration and the measurements at low exposures are the root cause of the fecundity over-prediction, highlighting a potential source of experimental noise. Higher predicted estradiol concentrations due to these two data points shifting the regressions would propagate through the model, resulting in higher predictions for vitellogenin concentration and average fecundity.

One way to improve predictive fidelity is to use source data in favor of summary statistics; in this example we have used the reported averages from multiple experiments. Effects from abnormally high or low data fluctuations should get washed away in the bootstrapped regressions easier when using individual data points rather than the averages. This case study, however, reveals the potential power of this modeling approach in highlighting experimental results that need increased resolution. By analyzing the model’s predictions against the experimental data, future experiments could be planned to further increase the accuracy of individual response-response regressions.

## Discussion

The veracity of our approach is illustrated using a case study spanning multiple scales of biological organization, from the molecular represented by receptor interactions, to the population scale with average fecundity. In particular, the results of thousands of simulations across increasing levels of input revealed thresholds of biological activity that partitioned each system into three distinct regions of no impact, moderate impact, or high impact on an apical endpoint measurement, to fathead minnow fecundity (reproduction-related AOP).

A lack of high-quality data is a pressing problem for modeling biological processes with any reasonable fidelity. In the AOP example, this “sparse data” problem is compounded not only by few measured input/output Key Event relationships, but also by few replicates per condition. For example, whole animal toxicological studies may only report data from as few as 3 biological replicates [29]. Our method can adapt to datasets with fewer biological replicates by increasing the number of iterations in the bootstrapping procedure (see Methods), but excels when questions arise regarding the degree of organism level effects can be associated with any of the individual Key Events in the AOP. In our given example, we use the average responses of plasma 17β-estradiol, plasma vitellogenin, and average fecundity, which were readily available in the literature. We assumed that the average responses represented an underlying normal distribution within the bulk data and captured the bootstrapped 95% confidence intervals for the fitted sigmoid parameters, which we then used to produce each individual run of the model. The final step is ultimately unnecessary, and was used in this work to highlight the distribution of response-response curves. The bootstrapped curves could be used instead as individual runs of the model, allowing the approach to capture the shape and modalities of any distributions present in the bulk data.

We used the fold-change in androgen receptor functionalization as the input data. If these inputs fall within the region we have associated with a moderate impact, then variation in the measured apical endpoints will be much more uncertain, because lower input values that infrequently lead to higher impacts will be conflated with higher input values with effects that are improbably screened out between levels of the hierarchy. More predictably, “lower” input values can be associated with a region of no predicted impact, whereas “higher” input values can be associated with impacts. As a result, this framework can be particularly useful in hazard screening and prioritization efforts by predicting the potential of chemicals to have an impact on a physiological scale (here fecundity) using data easily obtained from in vitro assays (e.g. estrogen receptor or androgen receptor activity) while accounting for biological variability across multiple scales. Given that there are tens of thousands of chemicals with little or no information on their potential environmental health effects, new modeling concepts such as ours are likely to have a great utility.

A primary assumption of the quantitative AOP model developed here is that Key Event molecules and proteins are organized into the same basic signaling system present within all individuals of the same species, age group, and gender. Although the considered population of fathead minnows is primarily composed of sexually mature females approximately 5–6 months old, it is not representative of wild populations that exhibit more physiological and habitat diversity. Thus, results from the AOP model are more limited and cannot directly inform reproductive toxicology or risk assessments of wild populations, which is a primary need driving AOP research in the field.

The case studies presented here provide insight into how information encoded in molecular fluctuations could propagate across a multiscale biological pathway. The network of Key Events associated with the AOP links a molecular initiating event to a macroscopic individual level outcome. In the AOP model, the output of one Key Event forms the input of the next, to form a chain of conditional influence between response fluctuations from a Key Event at a lower level of organization compound with those at a higher level. We might expect this system to produce a type of stochastic resonance, whereby larger (smaller) fluctuations compound and thus increase (decrease) the variance seen at higher levels of organization. However, the fecundity predictions illustrated in **Fig 3** are inconsistent with this expectation. Per Eqs **[1]** and **[2]**, interpolation of the dose-response data was carried out by curve-fitting to a sigmoid function, which fundamentally exhibits three regimes of response: two in which the response is constant for either “high” or “low” input values, and a power-law response that connects them at intermediate input values. That the dependence of fecundity on aromatase inhibition exhibits three clear regimes is a mathematical consequence of this compounding procedure: function iteration between sigmoids can be viewed as a Möbius transformation of the complex plane to yield another sigmoid—a fact that is independent of the number of function iterations/compounding steps. It follows from the sigmoid form that the effects of exceptionally large or small fluctuations in the input value will be quenched by virtue of falling within one of the two saturating regimes. The sigmoid relationship is phenomenologically ubiquitous across biology, possibly because it naturally arises from common occupancy driven processes such as receptor-ligand binding (e.g., see Ref. [30]).

Finally, the case study demonstrates the applicability of the method across various scales. The algorithm allows for the fast creation and simulation of models of biological systems based on existing experimental data. As systems biology evolves and models become more complex, development of these models will become more labor-intensive and more computationally expensive to run and analyze using traditional stochastic algorithms. The method thus represents an efficient means to simulate these larger systems. Future work will include expansion of the types of interactions (e.g. linear or non-monotonic relationships), the impact of multiple upstream signals converging on a single biomarker, and testing the algorithm on more complex multiscale systems.

## Supporting information

S1 Table (XLSX)

S2 Table (XLSX)
